# Comprehensive Pneumonitis Profile of Thoracic Radiotherapy Followed by Immune Checkpoint Inhibitor and Risk Factors for Radiation Recall Pneumonitis in Lung Cancer

**DOI:** 10.3389/fimmu.2022.918787

**Published:** 2022-06-20

**Authors:** Xiaotong Lu, Jianyang Wang, Tao Zhang, Zongmei Zhou, Lei Deng, Xin Wang, Wenqing Wang, Wenyang Liu, Wei Tang, Zhijie Wang, Jie Wang, Wei Jiang, Nan Bi, Luhua Wang

**Affiliations:** ^1^ Department of Radiation Oncology, National Cancer Center/National Clinical Research Center for Cancer/Cancer Hospital, Chinese Academy of Medical Sciences and Peking Union Medical College, Beijing, China; ^2^ Department of Diagnostic Radiology, National Cancer Center/National Clinical Research Center for Cancer/Cancer Hospital, Chinese Academy of Medical Sciences and Peking Union Medical College, Beijing, China; ^3^ Department of Medical Oncology, National Cancer Center/National Clinical Research Center for Cancer/Cancer Hospital, Chinese Academy of Medical Sciences and Peking Union Medical College, Beijing, China; ^4^ Department of Radiation Oncology, National Cancer Center/National Clinical Research Center for Cancer/Cancer Hospital and Shenzhen Hospital, Chinese Academy of Medical Sciences and Peking Union Medical College, Shenzhen, China

**Keywords:** immune checkpoint inhibitors, thoracic radiotherapy, checkpoint inhibitor pneumonitis, radiation pneumonitis, radiation recall pneumonitis, lung cancer, toxicity

## Abstract

**Purpose:**

Whilst survival benefits of thoracic radiotherapy (TRT) followed by immune checkpoint inhibitor (ICI) have been reported in patients with lung cancer, the potential high risk of treatment-related pneumonitis remains a concern. Asians may be more sensitive to lung toxicity than other races. This retrospective study intended to provide a comprehensive pneumonitis profile of TRT followed by ICI and investigate the risk factors from a Chinese cohort of lung cancer.

**Methods and Materials:**

From January 2016 to July 2021, 196 patients with lung cancer who received TRT prior to ICI were retrospectively analyzed. Treatment-related pneumonitis, including checkpoint inhibitor pneumonitis (CIP), radiation pneumonitis (RP), and radiation recall pneumonitis (RRP), were recorded and graded through medical records and chest computed tomography. Characteristics predictive of pneumonitis were assessed using logistic regression models, and the receiver operating characteristic analyses were performed to identify optimal cut points for quantitative variables.

**Results:**

With a median follow-up of 18 months, a total of 108 patients (55.1%) developed treatment-related pneumonitis during ICI therapy, with an incidence of 25.5% for grade 2 or higher (G2+) and 4.1% for G3+. The overall rates of CIP, RP and RRP were 8.2% (n=16), 46.9% (n=92) and 7.1% (n=14), respectively. With a total mortality rate of 1.5%, vast majority of the patients recovered from pneumonitis or remained stable. No patients died of RRP. Half of the patients with G2+ RP who withheld ICI therapy restarted ICI safely after resolution of RP. The history of chronic pulmonary diseases (*P*=0.05), mean lung dose (MLD, *P*=0.038), percent volume of lung receiving ≥5 Gy (V5, *P*=0.012) and percent volume of lung receiving ≥20 Gy (V20, *P*=0.030) predicted the occurrence of RRP in univariate analyses. Interval between TRT and ICI less than 3 months was an independent predictor for G2+ treatment-related pneumonitis in a multivariate model (Odds ratio OR=2.787, *P*=0.004).

**Conclusions:**

Treatment-related pneumonitis, especially RRP, is acceptable and manageable in the setting of TRT followed by ICI in this Asian population. Dosimetric parameters MLD, V5 and V20 may improve the predictions of RRP in clinical practice.

## Introduction

Immune checkpoint inhibitors (ICIs), which evoke an antitumor T-cell response by targeting programmed cell death 1/programmed cell death ligand 1 (PD-1/PD-L1) or cytotoxic T-lymphocyte-associated antigen 4 (CTLA-4), have advanced the treatment of lung cancer. According to the National Comprehensive Cancer Network (NCCN) guidelines for lung cancer (1, 2), ICIs have been recommended as the first-line or subsequent therapy options for both advanced non-small-cell lung cancer (NSCLC) and small-cell lung cancer (SCLC), and also as consolidation therapy for unresectable stage III NSCLC after definitive chemoradiotherapy.

Thoracic radiotherapy (TRT) is one of the most important treatment modalities for all stages of lung cancer. Preclinical studies have indicated a synergistic effect of radiation therapy and ICI, not only through the modulation of tumor immune microenvironment that sensitized the refractory “cold” tumor to immunotherapy (3), but also the “*in-situ* vaccine” effect that enhanced the immune response (4). Recent clinical trials, such as the landmark PACIFIC study (5), the LUNG 14-179 trial (6), and the STIMULI trial (7) showed significant survival benefits of TRT followed by ICI in patients with lung cancer. Nonetheless, as both TRT and ICI could provoke lung toxicity, the potentially high risk of treatment-related pneumonitis for the additive therapy remains a concern. A secondary analysis of KEYNOTE-001 indicated that NSCLC patients with previous TRT showed increased rate of any grade pneumonitis when received pembrolizumab (8). The PACIFIC study reported higher rates of both radiation pneumonitis (RP) and checkpoint inhibitor pneumonitis (CIP) in the durvalumab group when compared with the placebo group for stage III NSCLC patients after chemoradiation (9). Further, Asian patients were prone to develop treatment-related pneumonitis than others (10, 11).

TRT followed by ICI could induce various treatment-related pneumonitis, including CIP, RP and the special radiation recall pneumonitis (RRP). Yet, a comprehensive pneumonitis profile of TRT followed by ICI in Asian patients with lung cancer remains limited, and little is known about the ICI-related RRP. Hence, this single institution retrospective study intended to present the real-world incidences, characteristics, treatments, and outcomes of all kinds of treatment-related pneumonitis, including CIP, RP and RRP, in the setting of TRT followed by ICI from a Chinese cohort of lung cancer patients. Risk factors for symptomatic and severe treatment-related pneumonitis were investigated. We also assessed the risk factors for RRP in particular to help the clinicians understand this special pneumonitis in Asian patients better.

## Methods and Materials

### Patients and Treatments

Patients with pathologically confirmed lung cancer who received both radiotherapy and ICI at the Cancer Hospital, Chinese Academy of Medical Sciences from January 2016 to July 2021 were screened to identify those who received TRT prior to ICI. Patients with both ICI consolidation and subsequent ICI were included, and patients received antecedent ICI before TRT and the following ICI therapy were also eligible. Individual data of demography, tumor characteristics, treatment details, and dosimetry were collected under an institutional review board approved protocol. Chronic pulmonary diseases included chronic obstructive pulmonary disease, emphysema, interstitial lung disease, chronic bronchitis, asthma, pulmonary tuberculosis, and pulmonary hypoplasia. Biologically effective dose (BED) assuming an α/β of 10 for tumor was calculated to standardize the differences in dose and fractionation among all patients. MIM software was used to convert percent volume of lung receiving ≥5 Gy (V5), percent volume of lung receiving ≥20 Gy (V20), and mean lung dose (MLD) of different dose-fractionation schemes into 2 Gy equivalent doses assuming an α/β of 3 for normal lungs (12). Follow-up of chest computed tomography (CT) and symptom query *via* outpatient visit or phone call should be lasted at least 6 months after the onset of ICI.

### Pneumonitis Assessment

All types of treatment-related pneumonitis, including CIP, RP, and RRP, were identified separately by medical records and follow-up chest CT. The diagnosis of treatment-related pneumonitis was determined in consensus by the treating oncologists (W. J., Z. T., Z. Z., W. W., D. L., W. X., or L. W.) and two second radiation oncologists (L. X. and B. N.). Patients with controversial diagnoses of pneumonitis were discussed further in a multidisciplinary team including a medical oncologist, a radiologist and a radiation oncologist. The differentiation of CIP and RP was mostly based on the timing and CT characteristics of pneumonitis. RP usually occurred in less than 6 months after TRT within or at the edge of the radiation field, but CIP had a broader range of CT manifestations and longer time window. RRP was defined as inflammatory reactions at previously irradiated fields on chest CT after ICI administration and greater than 6 months after TRT (13). Acute exacerbation of preexisting radiation-induced lung fibrosis on chest CT after the commencement of ICI was also regarded as RRP. Pulmonary infections were ruled out by pathogenic microorganism detecting (e.g., sputum culture, or bronchoalveolar lavage culture if possible) and clinical suspicions of infection based on white blood cell count, C-reactive protein level, high fever, and CT characteristics. Furthermore, tumor progressions were also excluded by close follow-up or cytopathologic testing if possible.

Pneumonitis was graded using the Common Terminology Criteria for Adverse Events Version (CTCAE) 5.0. Grade 1 is radiographic changes confined to one lobe or less than 25% of the lung parenchyma without any symptom. Grade 2 is radiographic changes involved 25–50% of the lung parenchyma with mild symptoms that do not limit daily living. Grade 3 is indicated by severe respiratory symptoms that limit activities of daily living and radiographic changes involved all lung lobes or more than 50% of the lung parenchyma. Grade 4 is life-threatening symptoms that require urgent respiratory support, and grade 5 is death. For patients who developed treatment-related pneumonitis, date of pneumonitis diagnosis, CT characteristics, symptoms, managements and outcomes of pneumonitis were recorded through the medical records and telephone follow-ups. Recovery was defined as complete remission of symptoms as well as chest CT manifestations after clinical management of pneumonitis. Conversely, deterioration was defined as the aggravation of either symptoms or CT manifestations that led to death. Stabilization referred to a status between recovery and deterioration. The interval between the treatment and pneumonitis was defined as the time from the end of TRT or the commencement of ICI to the date of pneumonitis diagnosis.

### Statistical Analysis

Descriptive statistics were used to summarize the characteristics of patients, tumor and treatment details. These characteristics were assessed to predict for grade 2 or higher (G2+) treatment-related pneumonitis, G3+ treatment-related pneumonitis, and the development of RRP, respectively. Chi-squared tests or Fisher’s exact tests were generated for categorical variables and Mann-Whitney tests for continuous variables. Receiver operating characteristic (ROC) analyses were performed to identify optimal cut points for continuous variables. Cut points with maximal Youden Indexes that maximized the combined sensitivity and specificity were selected. Fitted regression models were used to calculate the probability of RRP at each dose value. Logistic regression model was used in multivariate analysis for G2+ treatment-related pneumonitis. Variables with P values less than 0.1 in univariate analyses and risk factors previously reported (i.e., age and smoking history) (11, 14) were included in the multivariate logistic regression model. Collinearity problem was comprehensively considered in model process as well. Due to the limited numbers of events, multivariate assessments were not performed for G3+ treatment-related pneumonitis or RRP. All analyses were performed using SPSS 26.0 and statistical significance was set at two-sided *P*<0.05.

## Results

### Patients and Treatment

Among 327 patients with lung cancer who received both radiotherapy and ICI, 196 patients who received TRT prior to ICI were analyzed. **Table 1** shows the demographics and clinical characteristics of these patients. With a median age of 61 years (range, 32-83), all patients had NSCLC (70.9%) or SCLC (29.1%). Most of these patients were male (81.7%); 94.4% of patients had an Eastern Cooperative Oncology Group performance status (ECOG PS) of 0 or 1; 73% of patients had a history of smoking; and 25.5% of patients had a history of chronic pulmonary diseases. All patients with adenocarcinoma (n=59) had molecular testing and 10 patients (5.1%) had EGFR activating mutations.

**Table 1:** Characteristics of patients, TRT and ICI therapy.

Characteristics	Classifications	Number (%) (n = 196)
Sex	Female Male	36 (18.3) 160 (81.7)
Median age, years (range)	61 (32-83)	–
ECOG PS	0-1 ≥2	185 (94.4) 11 (5.6)
History of smoking	No Yes	53 (27) 143 (73)
History of chronic pulmonary diseases	No Yes	146 (74.5) 50 (25.5)
Tumor histology	Adenocarcinoma Squamous cell lung carcinoma NSCLC-NOS SCLC	59 (30.1) 77 (39.3) 3 (1.5) 57 (29.1)
Genetic mutations	EGFR mutation With no mutation Unknown	10 (5.1%) 49 (25%) 137 (69.9%)
Initial cancer stage	I-II III IV	9 (4.6) 152 (77.6) 35 (17.9)
Lower lobe irradiation	No Yes	143 (73) 53 (27)
TRT types	Conventional Hypofractionated SBRT	184 (93.9) 9 (4.6) 3 (1.5)
Prior ICI therapy to TRT	No Yes	151 (77) 45 (23)
Concurrent chemoradiotherapy	No Yes	122 (62.2) 74 (37.8)
BED, Gy (range)	73.1 (39-115.2)	–
MLD, Gy (range)	11.3 (0.5-23.9)	–
Median V5, % (range)	39.4 (0.8-66.4)	–
Median V20, % (range)	19.1 (0-37)	–
ICI therapy types	Subsequent Consolidation	103 (52.6) 93 (47.4)
ICI agents	Durvalumab (PD-L1 inhibitor) Pembrolizumab Atezolizumab (PD-L1 inhibitor) Nivolumab Camrelizumab Toripalimab Sintilimab Tislelizumab Geptanolimab	51 (26) 29 (14.8) 18 (9.2) 15 (7.7) 27 (13.8) 24 (12.2) 24 (12.2) 6 (3.1) 2 (1)
ICI monotherapy	No Yes	123 (62.8) 73 (37.2)
Interval between TRT and ICI, days (range)	77 (0-1240)	–

ECOG PS, The Eastern Cooperative Oncology Group Performance Status; NSCLC-NOS, non-small cell lung cancer- not otherwise specified; SCLC, small cell lung cancer; TRT, thoracic radiotherapy; SBRT, stereotactic body radiation therapy; BED, biologically effective dose assuming an α/β of 10; MLD, mean lung dose; V5, percent volume of lung receiving ≥5 Gy; V20, percent volume of lung receiving ≥20 Gy; ICI, immune checkpoint inhibitor.

The majority of patients (93.9%) received conventional fractionated TRT, and 37.8% of patients received concurrent chemoradiotherapy. The median BED and MLD for all patients were 73.1 Gy (range, 39-115.2) and 11.3 Gy (range, 0.5-23.9), respectively. Median V5 and V20 were 39.4% (range, 0.8-66.4) and 19.1% (range, 0-37), respectively. All patients received PD-1 inhibitors (64.8%) or PD-L1 (35.2%) at a median interval of 77 days (range, 0-1240) after the end of TRT; 47.4% of patients received ICI consolidation while the other 52.6% received subsequent ICI after progressions or metastases; 37.2% of patients had ICI monotherapy while the other 62.8% had ICI combination therapy. Of note, there were 45 patients (23%) had antecedent ICI before TRT and the following ICI therapy. Among patients with SCLC (n=57), the numbers of limited-stage and extensive-stage SCLC patients were 42 (73.7%) and 15 (26.3%), respectively. Most of these patients (78.9%, n=45) received subsequent ICI therapy after progressions or metastases, while the rest of patients (21.1%, n=12) received ICI consolidation.

### Incidence and Characteristics of Treatment-Related Pneumonitis

With a median follow-up time of 18 months (interquartile range, 12.5-28.7) from TRT, a total of 108 patients (55.1%) developed treatment-related pneumonitis during ICI therapy, with an incidence of 25.5% (n=50) for G2+ and 4.1% (n=8) for G3+. **Tables 2**, **3** show the grades, characteristics, treatments and outcomes of three types of treatment-related pneumonitis. CIP occurred in 16 patients (8.2%) with a median time of 129 days (range, 0-554) after the first dose of ICI, and the incidences of G2+ and G3+ CIP were 7.1% and 3%, respectively. No relationship was found between time to onset of CIP from the commencement of ICI and its severity (**Supplementary Figure 1A**). For patients with G2+ CIP, all of them received corticosteroid treatment and 12/14 (87.5%) patients recovered from CIP, but 2 patients (14.3%) deceased. All patients with G2+ CIP discontinued ICI and one patient had ICI rechallenge after recovering from grade 2 CIP. **Supplementary Table 1** presents the characteristics of patients with co-existence of different treatment-related pneumonitis. It was noteworthy that half (7/14) of the patients with G2+ CIP had a history of RP.

**Table 2:** Characteristics of different types of pneumonitis.

Variables	CIP (n = 16)	RP (n = 92)	RRP (n = 14)
CTCAE grade, n (%) 1 2 3 4 5	2 (12.5) 8 (50) 4 (25) 0 (0) 2 (12.5)	55 (59.8) 35 (38) 1 (1.1) 0 (0) 1 (1.1)	11 (78.6) 2 (14.3) 1 (7.1) 0 (0) 0 (0)
CT characteristics, n (%) Ground glass opacity Consolidation Patchy Stripe Reticular	5 (26.3) 5 (26.3) 9 (47.4) 3 (15.8) 3 (15.8)	2 (1.6) 44 (34.6) 99 (78) 42 (33.1) 5 (3.9)	0 (0) 8 (42.9) 11 (78.6) 7 (50) 0 (0)
Interval between TRT and pneumonitis, days (range)	–	88.5 (0-248)	271 (188-630)
Interval between ICI and pneumonitis, days (range)	129 (0-554)	–	147 (49-588)

CIP, checkpoint inhibitor pneumonitis; RP, radiation pneumonitis; RRP, radiation recall pneumonitis; CTCAE, Common Terminology Criteria for Adverse Events; TRT, thoracic radiotherapy; ICI, immune checkpoint inhibitor.

**Table 3:** Characteristics of symptomatic pneumonitis.

Variables	G2+ CIP (n = 14)	G2+ RP (n = 37)	G2+ RRP (n = 3)
Symptoms, n (%) Fever Cough Short of breath Dyspnea	4 (28.6) 3 (24.1) 5 (35.7) 6 (42.9)	5 (13.5) 20 (54.1) 14 (37.8) 3 (8.1)	0 (0) 1 (33.3) 2 (67.7) 0 (0)
ICI suspension, n (%) Resumption of ICI	14 (100) 1 (7.1%)	28 (75.7) 14 (50%)	3 (100) 0 (0%)
Treatment, n (%) Corticosteroid Supportive treatment	14 (100) 0 (0)	32 (86.5) 5 (13.5)	2 (67.7) 1 (33.3)
Outcome, n (%) Recovery Stabilization Deterioration	12 (87.5) 0 (0) 2 (14.3)	28 (75.7) 8 (21.6) 1 (2.7)	2 (66.7) 1 (33.3) 0 (0)

G2+, grade 2 or higher; CIP, checkpoint inhibitor pneumonitis; RP, radiation pneumonitis; RRP, radiation recall pneumonitis; ICI, immune checkpoint inhibitor.

RP occurred in 92 patients (46.9%) and the incidences of G2+ and G3+ RP were 18.9% and 1%, respectively. Median time to RP onset was 88.5 days (range, 0-248) from the end of TRT, and G2 RP had a trend occurring slightly earlier than G1 RP but without statistical significance (**Supplementary Figure 1B**, *P*=0.068). For patients with G2+ RP, 32/37 (86.5%) patients received corticosteroid while the other 5 patients (all had grade 2 RP) received supportive treatment only. Thirty-six out of 37 patients (97.3%) recovered from G2+ RP or remained stable, and only 1 patient (2.7%) deceased even after active treatment. Twenty-eight patients (75.7%) with G2+ RP withheld ICI therapy, but half of them (14/28, all had grade 2 RP) renewed ICI treatment safely after resolution of RP.

RRP occurred in 14 patients (7.1%) and the incidences of G2+ and G3+ RRP were 1.5% and 0.5%, respectively. The median time between completion of TRT and RRP was 271 days (range, 188-630) and the median interval between the commencement of ICI and RRP was 147 days (range, 49-588). No relationship was identified between the time to onset of RRP from ICI or TRT and its severity (**Supplementary Figures 1C, D**). For patients with G2+ RRP, two received corticosteroid while the other one received supportive treatment only. All the 3 patients with G2+ RRP terminated the ICI treatment, and all of them recovered from RRP. There were 4 RRP patients had a history of RP and one RRP patient had concurrent CIP (**Supplementary Table 1**).

**Figure 1:**
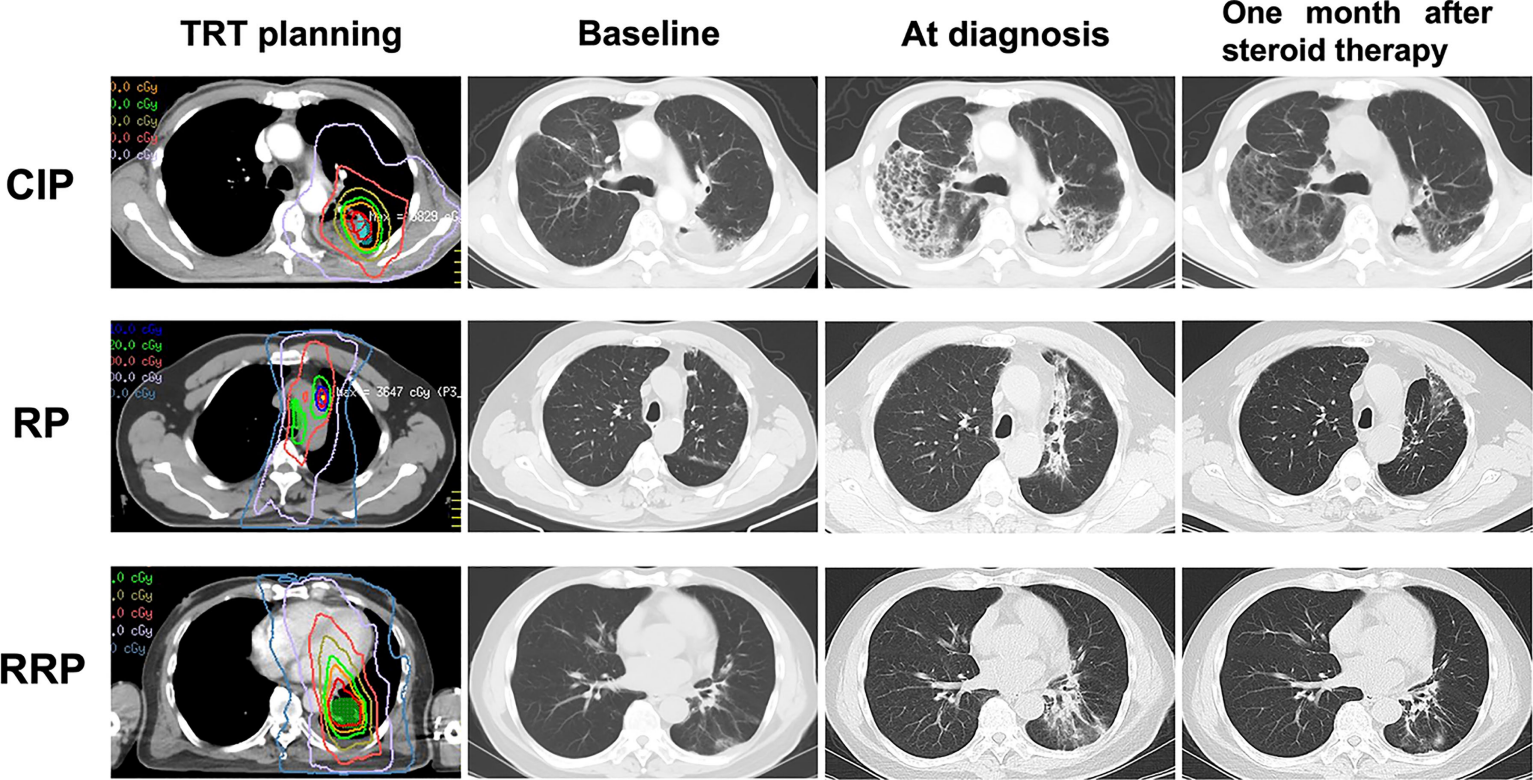
Distinctive CT changes of CIP, RP and RRP at baseline, at diagnosis and one month after steroid therapy. CIP, checkpoint inhibitor pneumonitis; RP, radiation pneumonitis; RRP, radiation recall pneumonitis; TRT, thoracic radiotherapy.

As displayed in **Table 2**, patients with RP and RRP had similar chest CT findings. About 78% of the patients presented as patches in the field of previous TRT. Consolidation and strip within the irradiation field were common for RP and RRP. More varied manifestations on chest CT were observed from patients with CIP than those with RP and RRP. Diffuse patchy (47.4%), consolidation (26.3%) and ground glass opacity (26.3%) were the three most frequent radiographic changes for CIP. CT changes of distinctive CIP, RP and RRP were presented in **Figure 1**. Cough and short of breath were the most common symptoms for RP and RRP while nearly half of the patients with symptomatic CIP had dyspnea, a severe respiratory symptom that required active management (**Table 3**). Fever was more common in patients with CIP (28.6%) than RP (13.5%) or RRP (0%). No patients with CIP, RP, or RRP complained of chest pain.

### Risk Factors for G2+ and G3+ Treatment-Related Pneumonitis

The results in **Table 4** demonstrate the associations between clinical, treatment, dosimetric factors and G2+ or G3+ treatment-related pneumonitis. On univariate analyses, age, female sex, smoking history, and dosimetric parameters (BED, MLD, V5, or V20) were not significantly associated with G2+ or G3+ treatment-related pneumonitis. In contrast, history of chronic pulmonary diseases (36% *vs.* 21.9%, *P*=0.049), ICI consolidation (62% *vs.* 42.5%, *P*=0.017) and interval between TRT and ICI less than 3 months (70% *vs.* 45.2%, *P*=0.002) were significantly associated with G2+ treatment-related pneumonitis when compared with the grade 0-1 group, but did not reach significance for G3+ group when compared with the grade 0-2 group. Notably, antecedent ICI therapy before TRT and the following ICI did not increase the incidence of G2+ or G3+ treatment-related pneumonitis. Multivariate analysis indicated that the interval between TRT and ICI less than 3 months (Odds ratio [OR]=2.787, 95% confidence interval [CI]: 1.394-5.572, P=0.004) was an independent risk factor for G2+ treatment-related pneumonitis. ICI consolidation was excluded from the multivariate model because of the collinearity (Cramer’s V value= 0.717, *P*< 0.001) with “interval between TRT and ICI less than 3 months”. Univariate analyses of risk factors for CIP and RP are shown in **Supplementary Tables 2**, **3**.

**Table 4:** The associations between clinical, treatment, dosimetric variables and G2+ or G3+ treatment-related pneumonitis.

Risk factors	G2+ *vs.* G 0-1 pneumonitis						G3+ *vs.* G 0-2 pneumonitis		
	Univariate analysis			Multivariate analysis			Univariate analysis		
	G2+ (n = 50)	G 0-1 (n = 146)	*P* value	OR	95% CI	*P* value	G3+ (n = 8)	G 0-2 (n = 188)	*P* value
Sex Male, n (%) Female, n (%)	39 (78) 11 (22)	121 (82.9) 25 (17.1)	0.526	–			8 (100) 0 (0)	152 (80.9) 36 (19.1)	0.355
Median age, years (range)	62.5 (32-73)	60 (37-83)	0.183	1.012	0.969-1.058	0.579	64.5 (54-67)	60 (32-83)	0.327
ECOG PS, n (%) 0-1 ≥2	48 (96) 2 (4)	137 (93.8) 9 (6.2)	0.733	–			8 (100) 0 (0)	177 (94.1) 11 (5.9)	1.000
Smoking history, n (%) No Yes	15 (30) 35 (70)	38 (26) 108 (74)	0.585	0.735 (yes vs. no)	0.345- 1.562	0.423	1 (12.5) 7 (87.5)	52 (27.7) 136 (72.3)	0.685
History of chronic lung diseases, n (%) No Yes	32 (64) 18 (36)	114 (78.1) 32 (21.9)	**0.049**	1.952 (yes vs. no)	0.953-3.995	0.067	4 (50) 4 (50)	142 (75.5) 46 (24.5)	0.116
Tumor histology, n (%) NSCLC SCLC	38 (76) 12 (24)	101 (69.2) 45 (30.8)	0.471	–			4 (50) 4 (50)	135 (71.8) 53 (28.2)	0.234
Lower lobe radiation, n (%) No Yes	37 (74) 13 (26)	106 (72.6) 40 (27.4)	1.000	–			5 (62.5) 3 (37.5)	138 (73.4) 50 (26.6)	0.448
Concurrent chemoradiotherapy, n (%) No Yes	26 (52) 24 (48)	96 (65.8) 50 (34.2)	0.083	1.498 (yes vs. no)	0.759- 2.954	0.244	4 (50) 4 (50)	103 (54.8) 85 (45.2)	1.000
Median BED, Gy (range)	73 (47-78)	73 (39-115)	0.767	–			73 (60-73)	73 (39-115)	0.852
Median MLD, Gy (range)	11.6 (4.2-17)	10.9 (0.5-23.9)	0.334	–			13.3 (7.6-14.4)	11.3 (0.5-23.9)	0.399
Median V5, % (range)	38.7 (12.1-59.4)	39.4 (0.8-66.4)	0.720	–			41.1 (25-54.6)	39.2 (0.8-66.4)	0.466
Median V20, % (range)	19.7 (6.6-28)	18.8 (0-37)	0.372	–			23 (11.9-25.9)	19.1 (0-37)	0.425
Antecedent ICI therapy, n (%) No Yes	38 (76) 12 (24)	113 (77.4) 33 (22.6)	0.847	–			7 (87.5) 1 (12.5)	144 (76.6) 44 (23.4)	0.685
ICI consolidation, n (%) No Yes	19 (38) 31 (62)	84 (57.5) 62 (42.5)	**0.017**	–			5 (62.5) 3 (37.5)	98 (52.1) 90 (47.9)	0.724
Interval less than 3 months between TRT and ICI, days (range) No Yes	15 (30) 35 (70)	80 (54.8) 66 (45.2)	**0.002**	2.787 (yes vs. no)	1.394- 5.572	**0.004**	4 (50) 4 (50)	91 (48.4) 97 (51.6)	1.000

G2+, grade 2 or higher; G3+, grade 3 or higher; OR, odds ratio; CI, confidence interval; ECOG PS, The Eastern Cooperative Oncology Group Performance Status; NSCLC, non-small cell lung cancer; SCLC, small cell lung cancer; BED, biologically effective dose assuming an α/β of 10; MLD, mean lung dose; V5, percent volume of lung receiving ≥5 Gy; V20, percent volume of lung receiving ≥20 Gy; TRT, thoracic radiotherapy; ICI, immune checkpoint inhibitor. Bold values imply P values with significant statistical difference.

### Risk Factors for Radiation Recall Pneumonitis

**Figure 2:**
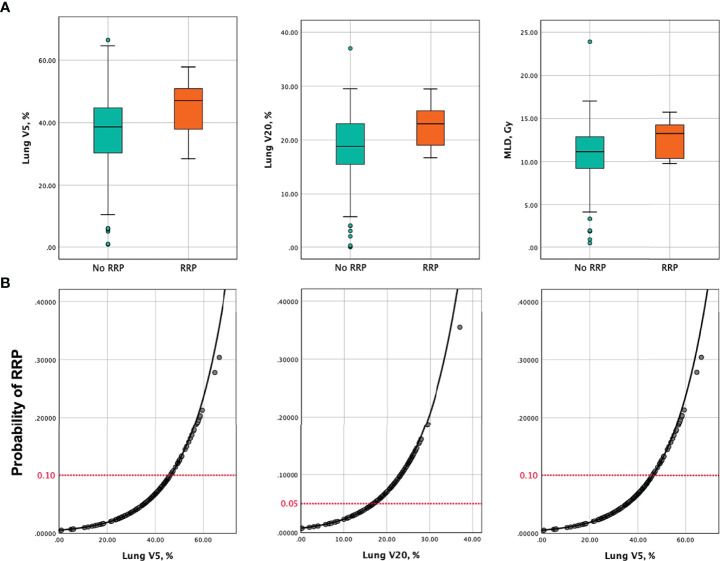
**(A)** Box and whisker plots of V5, V20 and MLD among patients who developed RRP versus those without. **(B)** Probability of developing RRP as a function of V5, V20, and MLD. Predictors of RRP are represented in separate models. The red horizontal dashed lines indicate the probability of RRP corresponding to the recommended dose metrics constraints in this analysis (V5 ≤ 46.3%, V20 ≤ 16.7%, MLD ≤ 12.9 Gy). V5, percent volume of lung receiving ≥5 Gy; V20, percent volume of lung receiving ≥20 Gy; MLD, mean lung dose; RRP, radiation recall pneumonitis.

The results of univariate analyses of risk factors for the development of RRP are shown in **Table 5**. From the univariate analyses, dosimetric parameters including MLD (13.2 Gy *vs.* 11.1 Gy, *P*=0.038), V5 (47% *vs.* 38.7%, *P*=0.012) and V20 (23% *vs.* 18.8%, *P*=0.030), were significantly higher among patients with RRP than those without (**Figure 2A**). ROC analyses found the optimal cutoffs of dosimetric parameters that predicted RRP, which corresponded to MLD > 12.9 Gy, V5 > 46.3%, V20 > 16.7%. The relationship of aforementioned dosimetric parameters and RRP was presented in **Figure 2B**, and the predicted RRP rates agreed with observed RRP events. Further, the history of chronic pulmonary diseases (50% vs. 23.6%, *P*=0.05) was marginally significant to predict the development of RRP. Smoking history, female sex, age, lower lobe irradiation, types of ICI therapy (monotherapy vs. combination therapy; PD-1 inhibitors vs. PD-L1 inhibitors), and interval between TRT and ICI were not associated with the occurrence of RRP. Of note, prior RP or CIP did not increase the risk of RRP development either.

**Table 5:** Univariate analysis of risk factors for the development of RRP.

Characteristics	RRP (n = 14)	No RRP (n =1 82)	*P* values
Sex Male, n (%) Female, n (%)	11 (78.6) 3 (21.4)	149 (81.9) 33 (18.1)	0.725
Median age, y (range)	60 (49-78)	61 (32-83)	0.617
ECOG PS, n (%) 0-1 ≥2	12 (85.7) 2 (14.3)	173 (95.1) 9 (4.9)	0.180
Smoking history, n (%) No Yes	3 (21.4) 11 (78.6)	50 (27.5) 132 (72.5)	0.762
History of chronic lung diseases, n (%) No Yes	7 (50) 7 (50)	139 (76.4) 43 (23.6)	**0.050**
Tumor histology, n (%) NSCLC SCLC	10 (71.4) 4 (28.6)	129 (70.9) 53 (29.1)	1.000
Lower lobe radiation, n (%) No Yes	11 (78.6) 3 (21.4)	132 (72.5) 50 (27.5)	0.762
Concurrent systemic therapy with TRT, n (%) No Yes	9 (64.3) 5 (35.7)	98 (53.8) 84 (46.2)	0.450
Median BED, Gy (range)	73 (60-78)	73 (39-115)	0.538
Median MLD, Gy (range)	13.2 (9.7-15.7)	11.1 (0.5-23.9)	**0.038**
Median V5, % (range)	47 (28.5-57.8)	38.7 (0.8-66.4)	**0.012**
Median V20, % (range)	23 (16.7-29.5)	18.8 (0-37)	**0.030**
Antecedent ICI therapy, n (%) No Yes	9 (64.3) 5 (35.7)	142 (78) 40 (22)	0.319
ICI monotherapy, n (%) No Yes	10 (71.4) 4 (28.6)	113 (62.1) 69 (37.9)	0.576
ICI agents, n (%) PD-1 inhibitors PD-L1 inhibitors	11 (78.6) 3 (21.4)	116 (63.7) 66 (36.3)	0.386
Interval between TRT and ICI therapy, days (range)	95 (4-433)	78 (0-1240)	0.760
Prior RP, n (%)	4 (28.6)	88 (48.4)	0.153
Concurrent CIP, n (%)	1 (7.1)	15 (8.2)	1.000

RRP, radiation recall pneumonitis; ECOG PS, The Eastern Cooperative Oncology Group Performance Status; NSCLC, non-small cell lung cancer; SCLC, small cell lung cancer; BED, biologically effective dose assuming an α/β of 10; MLD, mean lung dose; V5, percent volume of lung receiving ≥5 Gy; V20, percent volume of lung receiving ≥20 Gy; TRT, thoracic radiotherapy; ICI, immune checkpoint inhibitor; RP, radiation pneumonitis; CIP, checkpoint inhibitor pneumonitis. Bold values imply P values with significant statistical difference.

## Discussion

This study presented a comprehensive treatment-related pneumonitis profile of TRT followed by ICI from a Chinese cohort of lung cancer, which included the incidences, characteristics, treatments and outcomes of CIP, RP, and RRP. We found that the overall rates of all grade, G2+, G3+ treatment-related pneumonitis were 55.1%, 25.5% and 4.1%, respectively. With a total mortality rate of 1.5%, vast majority of the patients recovered from pneumonitis or remained stable, indicating the safety of ICI therapy after TRT in Asian patients. Of note, this is the first study that illustrates the predictive roles of dosimetric parameters (MLD > 12.9 Gy, V5 > 46.3%, V20 > 16.7%) for RRP. These modifiable factors may help oncologists assess the risk of RRP.

Lung cancer is not only the most common cancer but the leading cause of cancer death in China (15) and worldwide (16). In the era of immune therapy, ICI combined with TRT achieved striking survival benefits for both NSCLC and SCLC (5–7). As the most common high-grade and grade 5 adverse event (17), treatment-related pneumonitis deserves the uttermost attention in additive therapy of ICI and TRT. The incidences of any grade pneumonitis and G3+ pneumonitis in clinical trials of ICI after conventional TRT were 13-33% and 1-9%, respectively (6–9), whereas higher rates (56-62% and 2-14%, respectively) were observed in real-world data (11, 18–21). In Keynote 799, a phase 2 nonrandomized trial investigating the effect and safety of pembrolizumab plus concurrent CRT followed by consolidation pembrolizumab, the incidence of any grade treatment-related pneumonitis was 37.5% (22), which was lower than that of our study (CIP+RP, 55.1%) even with a high-intensive treatment. The possible reasons are as follows: First, Asian patients have significantly higher rate of all grade pneumonitis than those from Western countries in ICI consolidation after chemoradiotherapy according to subgroup analysis of PACIFIC trial (47.9% vs. 17.6%) and a real-world meta-analysis (62% vs. 22%, p=0.017) (10, 11). All patients were Chinese in our study but Keynote 799 trial only included about 10% Asian patients. Second, real-world incidence of lung toxicity is usually higher than that in clinical trials. Third, there might be differences in judgement of pneumonitis. However, it should be noted that the incidence of severe G3+ treatment-related pneumonitis in our study was comparable to those of other studies mentioned above, and also comparable to the results of concurrent chemoradiotherapy for stage III NSCLC at our institution (23), indicating the safety of ICI after TRT for Asians.

This study also presented the data of incidences, distinctive CT characteristics, treatments, and outcomes of CIP, RP and RRP in this Chinese cohort, providing a comprehensive pneumonitis profile in the real-world setting of TRT followed by ICI for lung cancer patients. Although the overall incidence of RP (46.9%) was much higher than that of CIP (8.2%), two thirds of RP patients were asymptomatic with no need of treatment while 80% of the CIP were symptomatic and required clinical intervention. For patients who developed pneumonitis, mortality rate was much higher in patients with CIP (12.5%) than those with RP (1.1%). These results indicated that more attention should be paid to patients with CIP, and more active treatment and closer monitoring are required. In line with the recommendation of ASCO guideline (24), all patients with G2+ CIP in this retrospective study discontinued ICI therapy and received corticosteroid, and 87.5% recovered. However, about one fifth of the patients with G2+ RP in our study received supportive therapy without corticosteroid also recovered from RP. Half of the patients with G2+ RP that had ICI interruption restarted ICI safely after resolution of RP, and all of them had grade 2 RP. Therefore, as the maintenance of ICI treatment benefits the survival (25), “over-reaction” may be avoided and the resumption of ICI should not be deterred for patients who developed grade 2 RP during ICI treatment after TRT.

RRP is known as a rare and delayed inflammatory reaction triggered by systemic drugs (e.g., chemotherapy agents and tyrosine kinase inhibitors) (26, 27) in previously irradiated area of lung. Yet, reports concerning the characteristics and risk factors of ICI-related RRP are limited. In this Chinese cohort, we observed an acceptable incidence of RRP (7.1%) and a low risk of G3+ RRP (0.5%), occurring as late as 2 years after TRT during ICI therapy of patients with lung cancer. The incidence of ICI-related RRP in our analysis seemed slightly higher than that triggered by chemotherapy (12 patients with RRP during 8 years) (26) or tyrosine kinase inhibitors (4.4%) (27). In a cohort of Japanese patients with NSCLC who received nivolumab, the incidence of RRP was 5.4% among 257 patients with history of previous TRT (28). However, in another retrospective study of Belgium patients with lung cancer, 15 out of 80 (18.8%) patients treated with ICI after TRT presented with RRP (13). This incidence was unexpectedly higher than that of Asians. Racial difference or diversity in the diagnosis of RRP, pending further validation, may account for the different results. Importantly, in line with the previous study (28), all patients in our cohort recovered from RRP without deterioration or death, supporting a satisfactory outcome of RRP.

Of note, we found significant associations between modifiable dosimetric variables (i.e. MLD, V5 and V20) and RRP in this study. Our results indicated that MLD, V5 and V20 predicted the occurrence of RRP. These modifiable risk factors might be useful for oncologists to evaluate the radiation therapy plan and identify the patients who are at a high risk of developing RRP during the following ICI treatment. Furthermore, advanced radiation technologies, such as deep inspiratory breath hold, margin reduction, proton therapy, and image-guided radiation therapy should be recommended to reduce the exposure of normal lung tissue in patients who plan to receive ICI after TRT (29). Although previous studies suggested a higher incidence of RRP among patients with previous CIP (13, 28), no significance was observed in our analysis. Due to the limited RRP events of all these studies, validation studies of larger-scale populations are necessary.

In our study, the interval between TRT and ICI less than 3 months was an independent predictor for G2+ treatment-related pneumonitis in a multivariate model, but not for G3+ group. Similarly, ICI consolidation improved the rate of G2+ pneumonitis due to the short interval between TRT and ICI when compared with subsequent ICI in univariate analysis. A recent pool analysis of trials in the US Food and Drug Administration Database showed a numerically higher rate of pneumonitis among patients received ICI within 3 months after radiotherapy when compared with those of more than 3 months, and the enhancement was mostly due to the low-grade pneumonitis (30). These results suggested that shorter interval between TRT and ICI increased low-grade but not severe treatment-related pneumonitis in lung cancer patients, which was acceptable and safe in the setting of additive therapy. As meta-analysis showed no survival difference between consolidative durvalumab within and beyond 42 days after TRT (11), a slight longer interval between TRT and ICI could be considered to balance the survival benefit and lung toxicity in the setting of ICI consolidation after chemoradiation for stage III NSCLC patients, especially for patients with risk factors predisposed to CIP or RP.

Previous studies have investigated the interaction between ICI-related pneumonitis and radiation-induced lung toxicity. Tamiya et al. reported that patients with a history of RP had a numerically higher rate of Nivolumab-related pneumonitis compared to those without RP (26.5% vs. 9.6%) (31). In our study, half of patients with G2+ CIP had a history of RP, indirectly supporting the results from Tamiya et al. On the other hand, Shaverdian et al. reported that patients with prior immune-related adverse events, such as CIP, were at very high risk for G2+ RP (61%) after TRT (29). It is convinced that the synergistic effect of TRT and ICI is strong, careful consideration and close monitoring of pneumonitis are needed for patients with previous treatment-related pneumonitis evoked by either TRT or ICI. However, we did not observe high incidence of pneumonitis among patients with antecedent ICI before TRT, while a small-size, single-arm retrospective study showed a relative high rate of G3+ RP (22.5%) in patients with prior ICI (32). Dosimetric variables were also not associated with G2+ or G3+ treatment-related pneumonitis in this study. Besides the limitation of sample size, one possibility was that the dose limits of normal lungs were relatively strict in this study. When bilateral lung doses were restricted to a low level, their predictive values might be controversial (33). In consist with prior studies (34), history of chronic pulmonary diseases increased the risk of not only G2+ treatment-related pneumonitis but also RRP in our results. Other potential factors, such as elder age (11) and non-smoker (14) were not significant in our analysis. Further studies with larger sample size and including biological biomarkers from next generation sequencing are warranted to shed more light on the prediction of treatment-related pneumonitis in patients treated with TRT and ICI in the future.

There are several limitations in our study. First, intrinsic limitations of retrospective analyses, such as selection bias, patient’s heterogeneities, and relatively finite sample size, were unavoidable from our study. Further large population confirmatory studies are warranted. Second, due to the diverse tumor histology and tumor stage, we were not equipped to investigate the relationship of treatment-related pneumonitis and the survival of lung cancer patients. Third, due to the variety of ICI agents and small numbers of patients with each ICI modality, we did not evaluate the influence of every single ICI agent for treatment-related pneumonitis. Last but not the least, the diagnoses of CIP, RP and RRP were subjective, and the differentiations among them were not easy. Some cases remained difficult to be differentiated from CIP and RP even after being discussed in an experienced multidisciplinary team. We tried our best to assess and grade the pneumonitis of every single patient and adopted the diagnoses with the most consensus of multidisciplinary teammates. These strategies helped provide relatively precise results of incidences and grades of CIP, RP and RRP, respectively.

## Conclusions

With a relatively high rate of all grade treatment-related pneumonitis, the occurrence of severe treatment-related pneumonitis and RRP are acceptable and manageable in patients with lung cancer who received ICI after TRT. Our study suggest that it is safe to start ICI therapy in less than 3 months after TRT as it increases the risk of low-grade but not severe pneumonitis. Notably, dosimetric parameters (i.e., V5, V20, and MLD) may help predict the development of RRP.

## Data Availability Statement

The original contributions presented in the study are included in the article/**Supplementary Material**. Further inquiries can be directed to the corresponding authors.

## Ethics Statement

The studies involving human participants were reviewed and approved by Cancer Hospital, Chinese Academy of Medical Sciences. The patients/participants provided their written informed consent to participate in this study.

## Author Contributions

XL reviewed the patients’ records, built the database and performed statistical analysis. JW, TZ, ZZ, LD, XW, WW, and WL assisted the records review and patients’ follow-up. XL and NB made the primary diagnosis of pneumonitis. WT, ZW, JW, WJ, and LW participated in the multidisciplinary discussion of treatment-related pneumonitis. XL wrote the article. NB and LW revised the article. All authors contributed to the article and approved the submitted version.

## Funding

This work was supported by the National Natural Sciences Foundation Key Program (81872474) and the Sanming Project of Medicine in Shenzhen (SZSM201612063).

## Conflict of Interest

The authors declare that the research was conducted in the absence of any commercial or financial relationships that could be construed as a potential conflict of interest.

## Publisher’s Note

All claims expressed in this article are solely those of the authors and do not necessarily represent those of their affiliated organizations, or those of the publisher, the editors and the reviewers. Any product that may be evaluated in this article, or claim that may be made by its manufacturer, is not guaranteed or endorsed by the publisher.
