# Supplementary material for: Comprehensive Pneumonitis Profile of Thoracic Radiotherapy Followed by Immune Checkpoint Inhibitor and Risk Factors for Radiation Recall Pneumonitis in Lung Cancer
Source: Front Immunol. 2022 Jun 20;13:918787. doi: 10.3389/fimmu.2022.918787 (PMC9251068; doi:10.3389/fimmu.2022.918787)
Supplement: Supplementary file 1 [file DataSheet_1.docx]

Supplementary Material

**Supplementary Table 1.** Individual characteristics of patients with co-existence of different treatment-related pneumonitis

| Patients | Age | PS | History of chronic lung diseases | Type of TRT | BED (Gy) | V20 (%) | V5 (%) | MLD (Gy) | ICI agents | Interval between TRT and ICI, days | Pneumonitis 1 | Date 1 | Pneumonitis 2 | Date 2 |
| --- | --- | --- | --- | --- | --- | --- | --- | --- | --- | --- | --- | --- | --- | --- |
| 1 | 59 | 1 | No | Conventional | 73.1 | 20.7 | 37.9 | 11.4 | Toripalimab | 42 | G2 RP | 2019/8/23 | G1 RRP | 2021/2/22 |
| 2 | 57 | 1 | Yes | Conventional | 67.9 | 25.9 | 49.3 | 14.2 | Nivolumab | 346 | G1 RP | 2019/11/28 | G1 RRP | 2020/9/4 |
| 3 | 70 | 1 | No | Conventional | 69.6 | 16.7 | 50.9 | 11.3 | Sintilimab | 146 | G2 RP | 2021/4/28 | G1 RRP | 2021/10/11 |
| 4 | 51 | 1 | Yes | Conventional | 73.1 | 25.4 | 47.4 | 13.9 | Pembrolizumab | 27 | G1 RP | 2020/12/7 | G1 RRP | 2021/7/21 |
| 5 | 58 | 1 | Yes | Conventional | 73.1 | 23 | 50 | 13.5 | Durvalumab | 189 | G3 CIP | 2020/12/20 | G3 RRP | 2020/12/20 |
| 6 | 67 | 1 | No | Conventional | 60 | 16 | 25 | 9.4 | Pembrolizumab | 301 | G1 RP | 2018/2/15 | G5 CIP | 2020/3/30 |
| 7 | 64 | 1 | Yes | Conventional | 73.1 | 23.1 | 54.6 | 13.5 | Sintilimab | 134 | G1 RP | 2019/9/4 | G3 CIP | 2019/12/29 |
| 8 | 60 | 1 | No | Conventional | 48 | 25.4 | 49.9 | 12.7 | Durvalumab | 49 | G2 RP | 2021/5/7 | G2 CIP | 2021/5/7 |
| 9 | 63 | 0 | No | Conventional | 73.1 | 17.7 | 37.4 | 10.8 | Durvalumab | 47 | G1 RP | 2021/4/7 | G2 CIP | 2021/10/23 |
| 10 | 46 | 0 | No | Conventional | 63.7 | 26 | 45 | 13.2 | Sintilimab | 165 | G1 RP | 2019/9/23 | G1 CIP | 2020/5/30 |
| 11 | 59 | 0 | Yes | Conventional | 60 | 28 | 45 | 13.2 | Tislelizumab | 318 | G1 RP | 2020/6/23 | G2 CIP | 2021/5/15 |
| 12 | 67 | 0 | Yes | Conventional | 73.1 | 24.5 | 53.9 | 14.4 | Durvalumab | 22 | G2 RP | 2021/2/26 | G3 CIP | 2021/5/1 |
| 13 | 64 | 1 | Yes | Conventional | 73.1 | 19.2 | 33 | 9.7 | Durvalumab | 79 | G2 RP | 2021/3/11 | G2 CIP | 2021/6/11 |
| 14 | 71 | 0 | No | Conventional | 73.1 | 18 | 43.7 | 11.3 | Durvalumab | 61 | G1 RP | 2021/9/8 | G1 CIP | 2021/12/31 |

**Abbreviations:** PS=performance score; TRT= thoracic radiotherapy; BED=biological equivalent dose assuming an α/β of 10; V20= percent volume of lung receiving ≥20 Gy; V5=percent volume of lung receiving ≥5 Gy; MLD=mean lung dose; ICI=immune checkpoint inhibitor; RP=radiation pneumonitis; CIP=checkpoint inhibitor pneumonitis; RRP=radiation recall pneumonitis.

**Supplementary Table 2.** Univariate analysis of risk factors for the development of CIP

| Characteristics | CIP (n=16) | No CIP (n=180) | *P* values |
| --- | --- | --- | --- |
| Sex  Male, n (%)  Female, n (%) | 15 (93.8)  1 (6.3) | 145 (80.6)  35 (19.4) | 0.313 |
| Median age, y (range) | 63.5 (43-71) | 60 (32-83) | 0.490 |
| ECOG PS, n (%)  0-1  ≥2 | 16 (100)  0 (0) | 169 (93.9)  11 (6.1) | 0.605 |
| Smoking history, n (%)  No  Yes | 2 (12.5)  14 (87.5) | 51 (28.3)  129 (71.7) | 0.244 |
| History of chronic pulmonary diseases, n (%)  No  Yes | 10 (62.5)  6 (37.5) | 136 (75.6)  44 (24.4) | 0.246 |
| Tumor histology, n (%)  NSCLC  SCLC | 10 (62.5)  6 (37.5) | 129 (71.7)  51 (28.3) | 0.566 |
| Lower lobe radiation, n (%)  No  Yes | 11 (68.8)  5 (31.3) | 132 (73.3)  48 (26.7) | 0.770 |
| Concurrent systemic therapy with TRT, n (%)  No  Yes | 6 (37.5)  10 (62.5) | 101 (56.1)  79 (43.9) | 0.193 |
| Median BED, Gy (range) | 72.4 (48-73.1) | 73.1 (39-115.2) | 0.104 |
| Median MLD, Gy (range) | 12.9 (7.6-14.6) | 11.3 (0.5-23.9) | 0.144 |
| Median V5, % (range) | 42.2 (25-54.6) | 39.2 (0.8-66.4) | 0.381 |
| Median V20, % (range) | 21.1 (11.9-28) | 19.1 (0-37) | 0.186 |
| Antecedent ICI therapy, n (%)  No  Yes | 13 (81.3)  3 (18.8) | 138 (76.7)  42 (23.3) | 1.000 |
| ICI monotherapy, n (%)  No  Yes | 10 (62.5)  6 (37.5) | 113 (62.8)  67 (37.2) | 0.982 |
| ICI agents, n (%)  PD-1 inhibitors  PD-L1 inhibitors | 8 (50)  8 (50) | 119 (66.1)  61 (33.9) | 0.196 |
| Interval between TRT and ICI therapy, days (range) | 106.5 (22-681) | 77 (0-1240) | 0.529 |
| RP before ICI therapy, n (%)  No  Yes | 11 (68.8)  5 (31.3) | 154 (85.6)  26 (14.4) | 0.142 |

**Abbreviations:** CIP=checkpoint inhibitor pneumonitis; ECOG PS=The Eastern Cooperative Oncology Group Performance Status; NSCLC=non-small cell lung cancer; SCLC=small cell lung cancer; BED= biologically effective dose assuming an α/β of 10; MLD=mean lung dose; V5=percent volume of lung receiving ≥5 Gy; V20= percent volume of lung receiving ≥20 Gy; TRT=thoracic radiotherapy; ICI=immune checkpoint inhibitor; RP=radiation pneumonitis.

**Supplementary Table 3.** Univariate analysis of risk factors for the development of G2+ RP

| Characteristics | G2+ RP (n=37) | G0-1 RP (n=159) | *P* values |
| --- | --- | --- | --- |
| Sex  Male, n (%)  Female, n (%) | 27 (73)  10 (27) | 133 (83.6)  26 (16.4) | 0.131 |
| Median age, y (range) | 61 (32-73) | 60 (37-83) | 0.328 |
| ECOG PS, n (%)  0-1  ≥2 | 35 (94.6)  2 (5.4) | 150 (94.3)  9 (5.7) | 1.000 |
| Smoking history, n (%)  No  Yes | 13 (35.1)  24 (16.8) | 40 (25.2)  119 (74.8) | 0.218 |
| History of chronic pulmonary diseases, n (%)  No  Yes | 25 (67.6)  12 (32.4) | 121 (76.1)  38 (23.9) | 0.284 |
| Tumor histology, n (%)  NSCLC  SCLC | 31 (83.8)  6 (16.2) | 108 (67.9)  51 (32.1) | 0.056 |
| Lower lobe radiation, n (%)  No  Yes | 28 (75.7)  9 (24.3) | 115 (72.3)  44 (27.7) | 0.680 |
| Concurrent systemic therapy with TRT, n (%)  No  Yes | 18 (48.6)  19 (51.4) | 89 (56)  70 (44) | 0.420 |
| Median BED, Gy (range) | 73.1 (47-76.2) | 73.1 (39-115.2) | 0.409 |
| Median MLD, Gy (range) | 11.4 (4.2-17) | 11.1 (0.5-23.9) | 0.952 |
| Median V5, % (range) | 38.5 (12.1-59.4) | 39.4 (0.8-66.4) | 0.972 |
| Median V20, % (range) | 19.7 (6.6-26.1) | 19 (0-37) | 0.716 |
| Antecedent ICI therapy, n (%)  No  Yes | 28 (75.7)  9 (24.3) | 123 (77.4)  36 (22.6) | 0.826 |
| ICI monotherapy, n (%)  No  Yes | 14 (37.8)  23 (62.2) | 109 (68.6)  50 (31.4) | **0.001** |
| ICI agents, n (%)  PD-1 inhibitors  PD-L1 inhibitors | 14 (37.8)  23 (62.2) | 113 (71.1)  46 (28.9) | **<0.001** |
| Interval between TRT and ICI therapy, days (range) | 41 (0-913) | 105 (1-1240) | **<0.001** |

**Abbreviations:** G2+ RP=grade 2 or higher radiation pneumonitis; G0-1: grade 0 and 1; ECOG PS=The Eastern Cooperative Oncology Group Performance Status; NSCLC=non-small cell lung cancer; SCLC=small cell lung cancer; BED= biologically effective dose assuming an α/β of 10; MLD=mean lung dose; V5=percent volume of lung receiving ≥5 Gy; V20= percent volume of lung receiving ≥20 Gy; TRT=thoracic radiotherapy; ICI=immune checkpoint inhibitor.


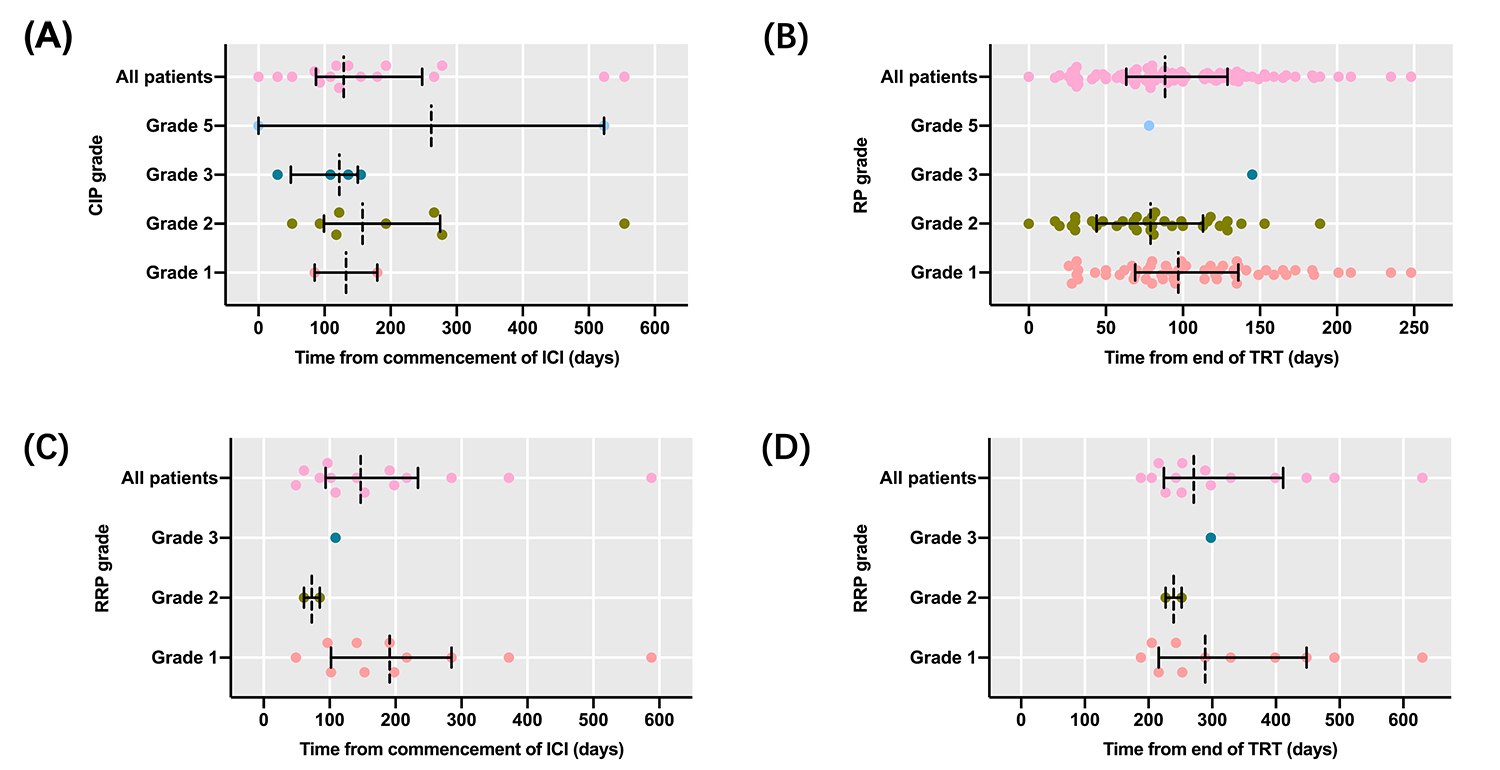


**Supplementary Figure 1.** Time to onset of treatment-related pneumonitis. (A) Time to onset of CIP from commencement of ICI; (B) Time to onset of CIP from commencement of ICI; (C) Time to onset of CIP from commencement of ICI; (D) Time to onset of CIP from commencement of ICI. Abbreviations: CIP=checkpoint inhibitor pneumonitis; ICI=immune checkpoint inhibitor; RP=radiation pneumonitis; TRT=thoracic radiotherapy; RRP=radiation recall pneumonitis.
